# Lung Cancer Detection Based on Kernel PCA-Convolution Neural Network Feature Extraction and Classification by Fast Deep Belief Neural Network in Disease Management Using Multimedia Data Sources

**DOI:** 10.1155/2022/3149406

**Published:** 2022-05-27

**Authors:** Deepak Kumar Jain, Kesana Mohana Lakshmi, Kothapalli Phani Varma, Manikandan Ramachandran, Subrato Bharati

**Affiliations:** ^1^Key Laboratory of Intelligent Air-Ground Cooperative Control for Universities in Chongqing, College of Automation, Chongqing University of Posts and Telecommunications, Chongqing, China; ^2^Department of Electronics and Communication Engineering, CMR Technical Campus, Kandlakoya, Secunderabad 501401, Telangana, India; ^3^Department of Electronics and Communication Engineering, Sagi Rama Krishnam Raju Engineering College, Bhimavaram 534204, Andhra Pradesh, India; ^4^School of Computing, SASTRA Deemed University, Thanjavur, India; ^5^Institute of Information and Communication Technology, Bangladesh University of Engineering and Technology, Dhaka 1205, Bangladesh

## Abstract

In lung cancer, tumor histology is a significant predictor of treatment response and prognosis. Although tissue samples for pathologist view are the most pertinent approach for histology classification, current advances in DL for medical image analysis point to the importance of radiologic data in further characterization of disease characteristics as well as risk stratification. Cancer is a complex global health problem that has seen an increase in death rates in recent years. Progress in cancer disease detection based on subset traits has enabled awareness of significant as well as exact disease diagnosis, thanks to the rapid flowering of high-throughput technology as well as numerous ML techniques that have emerged in recent years. As a result, advanced ML approaches that can successfully distinguish lung cancer patients from healthy people are of major importance. This paper proposed lung tumor detection based on histopathological image analysis using deep learning architectures. Here, the input image is taken as a histopathological image, and it has also been processed for removing noise, image resizing, and enhancing the image. Then the image features are extracted using Kernel PCA integrated with a convolutional neural network (KPCA-CNN), in which KPCA has been used in the feature extraction layer of CNN. The classification of extracted features has been put into effect using a Fast Deep Belief Neural Network (FDBNN). Finally, the classified output will give the tumorous cell and nontumorous cell of the lung from the input histopathological image. The experimental analysis has been carried out for various histopathological image datasets, and the obtained parameters are accuracy, precision, recall, and F-measure. Confusion matrix gives the actual class and predicted class of tumor in an input image. From the comparative analysis, the proposed technique obtains enhanced output in detecting the tumor once compared with an existing methodology for the various datasets.

## 1. Introduction

Cancer is a disease in which cells of the body multiply uncontrollably. When cancer spreads to the lungs, it is known as lung cancer. The leading cause of cancer is cigarette smoking. Tobacco, second-hand smoking, and occupational exposure to chemicals like asbestos or radon can cause lung cancer [1]. There are different types of lung cancer, and doctors can diagnose it using their procedures. To avoid human effort or error, we built a code in which we take a CT scan image and describe its properties, and then use various methods to determine whether the image is cancerous or not. Not only men but also women suffer from the same dangerous sickness in this world. Patient with lung cancer has a relatively short life expectancy after being diagnosed. CT scans from 61 patients were taken in Dicom format if the CT scans were taken in that format. There are 60 images in the database. We have presented a concept that reads JPEG-converted Dicom Format images of the lungs and uses image processing algorithms to look for any abnormalities. After the scanning is complete, the system calculates specific aspects of abnormality as well as feeds them into a system that has been taught to detect whether the anomaly is malignant. C 4.5 decision tree machine learning method is used for training. Conversion to grayscale, thresholding, histogram equalization, and feature extraction are among the image processing procedures. A total of 50 images are used to train the machine learning system. The result shows whether the tumor is cancerous or benign [2].

Millions of people are likely to benefit from the widespread adoption of lung cancer screening. However, radiologists face a significant workload due to the millions of CT scan pictures obtained from patients. Inter-rater disagreement has also been found [3]. Computer-aided diagnostic tools have already been shown to enhance the diagnosis of lung nodules in CT scans. Kaggle Data Science Bowl provided labelled chest CT images from 1397 patients to encourage the development of ML methods for automated CT diagnosis [4]. As a result, 1972 teams from all over the world have entered the competition, with 394 teams completing all phases, making it the largest healthcare-related Kaggle challenge ever. This is a once-in-a-lifetime opportunity to investigate the durability of medical ML methods as well as compare the performance of different methods for processing as well as categorising chest CT data at scale [5].

This paper proposes lung cancer detection using deep learning architectures for histopathology image analysis. The contributions made in this paper are as follows:

- To process the input histopathology image for noise removal, images resize, and normalize images.
- To extract the features of processed lung image using KPCA–CNN.
- To classify extracted features using FDBNN. The classified output shows the normal cell and cancerous cells of the input histology image.
- The simulation analysis has been carried out for various data sets for histopathology images for parametric analysis in terms of accuracy, precision, recall, and F-measure. By confusion matrix, the actual class, as well as the predicted class of tumor, has been shown.

Paper organization is as follows: related works are detailed in Section 2.  Section 3 defines the proposed methodology of lung cancer detection. Experimental analyses are in Section 4 and finally concludes in Section 5.

## 2. Related Works

Deep learning-based neural networks have emerged as the dominant technique in medical image analysis in recent years, outperforming algorithms based on handcrafted features. To variate lung cancer nodules from non-nodules, authors in [6] compared various methods. They developed the 3D Convolutional Neural Network Technique to minimize/eliminate false absolute predictions. False detections are obtained due to nodules in assorted sizes and utilizing one CNN. To find lung cancer using CT images, authors in [7] used 3D DenseNet and neural networks C3D. These NN are used in whole lung 3D images as well as two-stage methods (two distinct NN are trained for segmentation and classification) and compared. To determine malignancy level and segmentation are two architectures proposed in [8]. In [9], author proposed an automatic lung nodule detection module. A public data set called LIDC-IRDI exists used for the proposed method. Multi-Scene Deep Learning Framework is the proposed method. To detect lung cancer, CNN-based method is proposed in [10]. From 69 different patients, they consider only 100 images that are 50 others and 50 cancerous. Augmentation was utilized to get a healthy data set based on a fewer number of images. This study used VGG-16 CNNs, AlexNet, and LeNet. In [11], an iterative voting technique based on radial symmetries that are adaptable to perturbation as well as analyzing elliptical shapes based on this notion of radial symmetry-based voting is suggested. An iterative process is computationally costly. To solve this issue, the authors of [12] suggested single-pass voting for cell identification, in which only one round of voting is performed as well as final cell centers are computed by applying mean shift clustering [13] to the vote image. DL methods, particularly CNNs, have recently sparked interest and attained state-of-the-art performance in a variety of vision tasks, including image classification, object recognition, and segmentation [14]. The use of CNN in medical image analysis is claimed to be a huge success [15]. Deep CNN is used in histology breast cancer images for automatic mitotic cell detection [16]. The authors used CNN to compute pixel-wise coarse segmentation before applying a fast min-cut/max-flow graph inference technique to get the final nucleus positions [17]. The CNN models are used in both [18, 19] to test pictures in a sliding window fashion for pixel-wise classification, which is costly. FCN (Fully Conventional Network) was proposed in [20]. Unlike traditional CNN techniques [21], FCN is trained from beginning to finish and may create output maps same size as inputs, making it both asymptotically as well as absolutely efficient [22]. Despite the fact that several methodologies for lung cancer diagnostics is proposed in literature, these techniques have little potential for early cancer identification. The majority of these methods have a number of disadvantages, including excessive complexity, inability to provide acceptable results due to a lack of consideration of informative or relevant features as a goal, as well a higher number of iterations needed to achieve acceptable results.

## 3. Proposed Tumor Detection Model

**Figure 1:**
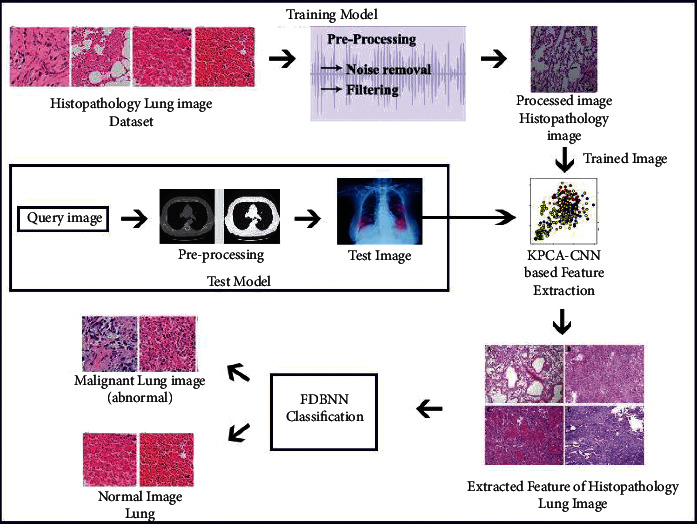
Overall system architecture.

This section discusses the model of lung cancer detection using deep learning technique-based architecture. Here, we use histopathological-based image analysis for lung cancer detection. The overall architecture of the proposed model is given in Figure 1.

Feature extraction using Kernel PCA integrated with convolutional neural network:

Kernel PCA is a PCA extension that uses kernels to allow for the separation of nonlinear data. The core notion is to project linearly inseparable data onto a higher dimensional region where it may be separated linearly. Kernel PCA projects a data set into a higher dimensional feature space, where it can be linearly separated, using a kernel function. Here extraction of input histology image has been carried out using KPCA-CNN. In HD space using kernel function, KPCA is a method for producing traditional linear PCA. Through some nonlinear, *u*(*xt*), *z* = *u*(*xt*). Let *H* be a Hilbert space that realizes trace class *G* as a self-adjoin operator. *T* ∈ *B* (*H*) is tumor trace class if and only if the series $\sum_{\text{set}}\left(\psi_{i},\left|T\right|\psi_{i}\right),\text{with}\left|\bar{T}\right|=\sqrt{T^{*}T},$ is convergent for some ONB *ψ*_*i*_. In this case, from (1),

(1) $$\begin{array}{c} tr\left(T\right)≔\sum \left(\psi_{i},T\psi_{i}\right),\\ c_{1}{\left\|f\right\|}^{2}\leq \underset{\alpha \in A}{\sum }{\left|\langle h_{\alpha },f\rangle \right|}^{2}\leq c_{2}{\left\|f\right\|}^{2}\text{for all }f\in ℋ. \end{array}$$

Let (*h*_*α*_)_*α*∈*A*_ be a frame in H. Set *L* : *H*⟶*l*^2^, then by

(2) $$\begin{array}{c} L:f⟷{\langle h_{\alpha },f\rangle }_{\alpha \in A}. \end{array}$$

Then *L∗* : *l*^2^⟶*H* given by

(3) $$\begin{array}{c} L^{*}\left(\left(c_{\alpha }\right)\right)=\sum_{\alpha \in A}c_{\alpha }h_{\alpha }, \end{array}$$

where (*c*_*α*_) ∈ *l*^2^; and by

(4) $$\begin{array}{c} L^{*}L=\underset{\alpha \in A}{\sum }|h_{\alpha }\rangle \langle h_{\alpha }|. \end{array}$$

A positive definite kernel on S is a function K : *S* × *S* ⟶ C, such that from (5),

(5) $$\begin{array}{c} \sum_{i,\;j=1}^{N}\bar{c_{i}}c_{j}K\left(v_{i},v_{j}\right)\geq 0. \end{array}$$

For all *|*{*x*_*i*_}_*i*=1_^*N*^ ⊂ *S*, {*c*_*i*_}_*i*=1_^*N*^ ⊂ ℂ, and *N* ∈ ℕ

Given a p.d. kernel as there exists RKHS *H* (*K*) and a mapping Φ: *S* ⟶ *H* (*K*) such that from (6),

(6) $$\begin{array}{c} K\left(x,y\right)={\langle \Phi \left(x\right),\Phi \left(y\right)\rangle }_{ℋ\left(K\right)}. \end{array}$$

For this issue, Φ is called a feature map. Further, reproducing property is given as

(7) $$\begin{array}{c} f\left(x\right)={\left(K_{x},f\right)}_{ℋ\left(K\right)}. \end{array}$$

For all *f* ∈ *H* (*K*), and *x* ∈ *S*. *H* (*K*) may be chosen as the Hilbert completion by (8):

(8) $$\begin{array}{c} \text{span}\left\{K_{x}≔K\left(.,x\right)\right.. \end{array}$$

*H* (*K*) is inner product is given by (9)

(9) $$\begin{array}{c} {\sum c_{i}K_{x_{i}},\sum d_{j}K_{x_{j}ℋ\left(K\right)}≔\sum {\bar{c}}_{i}d_{j}K\left(x_{i},x_{j}\right).}^{} \end{array}$$

A combination of pool and convolution layers is usually used in CNN architecture. Means and max-pooling are the two types of operation in the pooling layer. The average neighborhood is determined inside the feature points in mean pooling and within a maximum of feature points in max pooling. Mean pooling lowers the inaccuracy caused by the neighborhood size restriction while preserving background data.

Convolutional, max pooling, fully connected layers, and softmax functions are four types of layers. A 224 × 224 × 3 image is used as NNs input. Filters are 3D matrices with a stride of one. CNN is a set of convolutional as well as pooling layers that allow for the extraction of key characteristics from images that best match the end goal. Convolution is defined as a product on a volume by creating a convolution product on a 2D matrix, which is the sum of element-wise products. Based on chain rule and vector calculus, the CNN learning process is done.

Suppose *z* is a scalar and the vector is *y* ∈ ℝ^*H*^. A partial derivative of *z* based on *y* is a vector if *z* is a function of *y* is given by (10)

(10) $$\begin{array}{c} {\left[\frac{\partial z}{\partial y}\right]}_{i}=\frac{\partial z}{\partial y_{i}}. \end{array}$$

Consider, *x* ∈ ℝ^*w*^ is another vector and *y* is a function of *x*. With respect to *x*, partial derivative of *y* is given in (11)

(11) $$\begin{array}{c} {\left[\frac{\partial y}{\partial x^{T}}\right]}_{ij}=\frac{\partial y_{i}}{\partial x_{j}}. \end{array}$$

It is an *H* × *W* matrix, *i*th row and *j*th column entry at intersection level is *∂y*_*i*_/*∂x*_*j*_. Image with H rows, W columns, and 3 channels are the input of order 3 tensor which CNN considers. A layer is a type of processing step that is anything from a convolution layer to a pooling layer to a normalizing layer to a fully linked layer to a loss layer.

Input *x*^1^, make it pass processing of 1^st^layer, and get *x*^2^. In turn, *x*^2^ is passed into the 2^nd^ layer. Achieve *x*^*L*^ ∈ ℝ^*c*^, which evaluates **x**^*l*^ posterior probabilities belonging to *C* categories. CNN output prediction by (12)

(12) $$\begin{array}{c} \underset{i}{\text{arg max}}x_{i}^{L}. \end{array}$$

Already evaluated terms *∂z*/*∂ ***w**^*i*+1^and*∂z*/*∂ ***x**^*i*+1^. Compute *∂z*/*∂ ***w**^*i*^and*∂z*/*∂ ***x**^*i*^, using chain rule in (13)

(13) $$\begin{array}{c} \frac{\partial z}{\partial \left(\text{vec}{\left(w^{i}\right)}^{T}\right)}=\frac{\partial z}{\partial \left(\text{vec}{\left(x^{i+1}\right)}^{T}\right)}\frac{\partial \text{vec}\left(x^{i+1}\right)}{\partial \left(\text{vec}{\left(w^{i}\right)}^{T}\right)},\\ \frac{\partial z}{\partial \left(\text{vec}{\left(x^{i}\right)}^{T}\right)}=\frac{\partial z}{\partial \left(\text{vec}{\left(x^{i+1}\right)}^{T}\right)}\frac{\partial \text{vec}\left(x^{i+1}\right)}{\partial \left(\text{vec}{\left(x^{i}\right)}^{T}\right)}. \end{array}$$

Consider 1-th layer, whose inputs are an order of 3 tensors **x**^*l*^ with **x**^*l*^ ∈ ℝ^*H*^*l*^×*W*^*l*^×*D*^*l*^^. To designate any certain element in *x*^*l*^, triplet index set *i*^*l*^, *j*^*l*^, *d*^*l*^ is required. Triplet *i*^*l*^, *j*^*l*^, *d*^*l*^ indicates to one element in **x**^*l*^, which is in **d**^*l*^th channel, at spatial location (*i*^*l*^, *j*^*l*^). Mini batch method is utilized in actual CNN learning. **x**^*l*^ Becomes an order 4 tensor in ℝ^*H*^*l*^×*W*^*l*^×*D*^*l*×*N*^^ in which N is mini-batch size. Consider *N* = 1 for simplicity. Zero-based indexing convention is utilized to simplify notations that indicate 0 ≤ *i*^*l*^ < *H*^*l*^, 0 ≤ *j*^*l*^ < *W*^*l*^, and 0 ≤ *d*^*l*^ < *D*^*l*^.

In *l*th layer, a function will transfer input **x**^*l*^ to an output *y*, which is also input to the forthcoming layer. *y* and **x**^*l*+1^ in fact refers to the same object, and it is very helpful to keep this point in mind. Assume output with size *H*^*l*+1^ × *W*^*l*+1^ × *D*^*l*+1^ and an element in indexed output by a triplet, as shown in (14)

(14) $$\begin{array}{c} \left(i^{l+1},j^{l+1},d^{l+1}\right),\;0\leq i^{l+1}<H^{l+1},\;0\leq j^{l+1}<W^{l+1},\\ 0\leq d^{l+1}<D^{l+1}. \end{array}$$

**x** ^*l*^ and *y* share similar size, ReLU layer does not change input size. For every element in input, ReLU is considered as truncation that is given in (15)

(15) $$\begin{array}{c} y_{i,j,d}=\text{m}\left\{0,x_{i,j,d}^{l}\right\}. \end{array}$$

With 0 ≤ *i* < *H*^*l*^=*H*^*l*+1^, 0 ≤ *j* < *W*^*l*^=*W*^*l*+1^, and 0 ≤ *d* < *D*^*l*^=*D*^*l*+1^

In this layer, there is no need for parameter learning and no parameter inside the ReLU layer. Based on the above equation, it is obvious, as shown in (16)

(16) $$\begin{array}{c} \frac{\text{d}y_{i,j,d}}{\text{d}x_{i,j,d}^{l}}=\left[x_{i,j,d}^{l}>0\right]. \end{array}$$

[[.]] is denoted as an indicator function, and it is given in equation (17)

(17) $$\begin{array}{c} {\left[\frac{\partial z}{\partial x^{l}}\right]}_{i,j,d}=\left\{\begin{array}{cc} {\left[\frac{\partial z}{\partial y}\right]}_{i,j,d}, & \text{if }x_{i,j,d}^{l}>0\\ 0, & \text{otherwise}. \end{array}\right. \end{array}$$

Consider 2 × 2 convolution kernel size and 3 × 4 input image by combining a matrix with one single convolution kernel. Multiple convolution kernels are utilized in the convolution layer. Consider *D* kernels are utilized and every kernel is of spatial span *H* × *W*, *f* is an order 4 tensor in ℝ^*H*^*l*^×*W*^*l*^×*D*^*l*^^. Similarly, index variables 0 ≤ *i*^*l*^ < *H*^*l*^, 0 ≤ *j*^*l*^ < *W*^*l*^, and 0 ≤ *d*^*l*^ < *D*^*l*^ used to pinpoint a certain element in kernels. Consider stride is 1, and no padding is utilized. Hence, *y* or (*x*^*i*+1^) in ℝ^*H*^*H*+1^×*W*^*i*+1^×*D*^*t*+1^^ with *H*^*l*+1^=*H*^*l*^ − *H*+1, *W*^*l*+1^=*W*^*l*^ − *W*+1, and *D*^*l*+1^=*D*_2_, the convolution process is given in the equation: for all 0 ≤ *d* ≤ *D*=*D*^*l*+1^ and for any spatial location (*i*^*l*+1^, *j*^*l*+1^) satisfying $|0\leq i^{l+1}<{\bar{H}}^{l}-\bar{H}+1=H^{l+1},0\leq j^{l+1}<W^{l}-W+1=W^{l+1}$. In this equation, **x**^*l*^(*i*^*l*+1^, *j*^*l*+1^) refers to an element of **x**^*l*^ indexed by triplet (*i*^*l*+1^+*i*, *j*^*l*+1^+*j*, *d*^*l*^). *b*^*d*^ is bias term that is usually added to *y*(*i*^*l*+1^, *j*^*l*+1^). Consider **x**^*l*^ is a 3^rd^ order tensor in ℝ^*H*^*l*^×*W*^*l*^×*D*^*l*^^ with one element in **x**^*l*^ being indexed by a triplet (*i*^*l*^, *j*^*l*^, *d*^*l*^). Consider *f* is a set of convolution kernels with spatial extent are *H* × *W*. Converts **x**^*l*^ into a matrix ∅**x**^*l*^ by expansion operator. In this matrix, index elements are (*p*, *q*). Expansion operator copies element at (*i*^*l*^, *j*^*l*^, *d*^*l*^) in **x**^*l*^ to (*p*, *q*)th entry in ∅**x**^*l*^ From the definition of expansion procedure with (p, q), evaluate its corresponding (*i*^*l*^, *j*^*l*^, *d*^*l*^) triplet is *p*=*i*^*l*+1^+(*H*^*l*^ − *H*+1) × *j*^*l*+1^,  *q*=*i*+*H* × *j*+*H* × *W* × *d*^*l*^,  *i*^*t*^=*i*^*l*+1^+*i*,  *j*^*l*^=*j*^*i*+1^+*j*

Applying many filters on input with *ψ* is an activation function, where the convolution products are utilized at the convolution layer represented in (18).

(18) $$\begin{array}{c} \forall n\in \left[1,2,\ldots ,n_{C}^{\left[l\right]}\right]:\\ \text{con}v{\left(a^{\left[l-1\right]},K^{\left(n\right)}\right)}_{x,y}=\psi^{\left[l\right]}{\left(\sum_{i=1}^{n_{H}^{\left.n-1\right]}}\sum_{j=1}^{n_{W}^{\left.l-1\right]}}\sum_{k=1}^{n_{C}^{\left.l-1\right]}}K_{i,j,k}^{\left(n\right)}a_{x+i-1,y+j-1,k}^{\left[l-1\right]}+b_{n}^{\left[l\right]}\right)}_{\text{dim}\left(\text{conv}\left(a^{\left.l-1\right]},K^{\left(n\right)}\right)\right)=\left(n_{H}^{\left[l\right]},n_{W}^{\left[l\right.}\right)}. \end{array}$$

Thus by (19):

(19) $$\begin{array}{c} a^{\left[l\right]}=\psi^{\left[l\right]}\left(\text{conv}\left(a^{\left[l-1\right]},K^{\left(1\right)}\right)\right)\psi^{\left[l\right]}\left(\text{conv}\left(a^{\left[l-1\right]},K^{\left(2\right)}\right)\right),\\ ,\ldots ,\psi^{\left[l\right]}\left(\text{conv}\left(a^{\left[l-1\right]},K^{\left(n_{C}^{\left[l\right)}\right)}\right)\right). \end{array}$$

With (20):

(20) $$\begin{array}{c} n_{H/W}^{\left[l\right]}=\left[\frac{n_{H/W}^{\left(l-1\right)}+2p^{\text{'}l}-f^{ll}}{s^{ll}}+1\right];\;s>0\\ =n_{H/W}^{\left[l-1\right]}+2p^{\left[l\right]}-f^{\left[l\right]};\;s=0\\ n_{C}^{\left[l\right]}=\text{number of filters.} \end{array}$$

Slide a filter across an image, with no parameters in a particular stride, and apply a function given by (21)

(21) $$\begin{array}{c} \text{dim}\left(\text{pooling}\left(\text{image}\right)\right);\;s>0,\\ =\left(n_{H}+2p-f,n_{W}+2p-f,n_{\text{C}}\right);\;s=0\\ =\left(\left\lfloor \underline{n_{H}}+2p-fs+1\right\rfloor ,\left\lfloor \frac{n_{W}+2p-f}{s}+1\right\rfloor ,n_{C}\right). \end{array}$$

A fully connected layer is made up of a few neurons that take one vector as input and output another vector.

In general, considering *j*^th^ node of *i*^th^ layer by (22):

(22) $$\begin{array}{c} z_{j}^{\left[i\right]}=\overset{n_{i-1}}{\underset{l=1}{\sum }}w_{j,l}^{\left[i\right]}a_{l}^{\left[i-1\right]}+b_{j}^{\left[i\right]}\\ ⟶a_{j}^{\left[i\right]}=\psi^{\left[i\right]}\left(z_{j}^{\left[i\right]}\right). \end{array}$$

Input *a*^[*i* − 1]^ might be the solution of convolution or a pooling layer with dimensions (*n*_*H*_^[*i* − 1]^, *n*_*W*_^[*i* − 1]^, *n*_*C*_^[*i* − 1]^). Tensor flatten to a 10 vector of dimension in order to plug it into a fully linked layer: (*n*_*H*_^[*i* − 1]^ × *n*_*W*_^[*i* − 1]^ × *n*_*C*_^[*i* − 1]^, 1), thus from (23):

(23) $$\begin{array}{c} n_{i-1}=n_{H}^{\left[i-1\right]}\times n_{W}^{\left[i-1\right]}\times n_{C}^{\left[i-1\right]}. \end{array}$$

To determine lung tumors in CT image size 5 × 20 × 20, the backpropagation method is utilized to teach CNN. It is divided into two parts. To extract expedient volumetric characteristics from input data, a CNN with multiple ReLU, volumetric convolution, and max-pooling layers are engaged in 1^st^ phase. For tumor features, 2^nd^ phase is extraction. It has several FC and threshold layers by a SoftMax layer, which performs the neural network's high-level reasoning. To protect the original values of DICOM images are possible, no scaling used to remain conducted to the data set's histopathological images. During training, random subvolumes selected from the training set's histopathological images are normalized using an approximation of the normal distribution of the data set's voxel values.

Classification using Fast Deep Belief Neural Network (FDBNN):

FDBNN is a stacked RBM-based generative graphics model. DBN can capture a hierarchical representation of incoming data thanks to its deep structure. RBM is an energy-based probabilistic method that is a log-linear Markov Random Field and is a limited form of Boltzmann machines (BM). The input is represented by visible nodes *x*, while the latent characteristics are represented by hidden nodes *h*. Visible nodes joint distribution *x* ∈ ℝ^*J*^ and hidden variable *h* ∈ ℝ^*I*^ are given in (24)

(24) $$\begin{array}{c} p\left(x,h\right)=\frac{1}{Z}e^{-E\left(x,h\right)},E\left(x,h\right)=-hWx-ch-bx, \end{array}$$

where *W* ∈ ℝ^*I*×*J*^, *b* ∈ ℝ^*J*^, and*c* ∈ ℝ^*I*^ are model parameters and partition function is Z. Following form of conditional distributions by (25) since units in a layer are independent in RBM:

(25) $$\begin{array}{c} p\left(h|x\right)=\prod_{i=1}^{l}p\left(h_{i}|x\right),p\left(x|h\right)=\prod_{j=1}^{J}p\left(x_{j}|h\right). \end{array}$$

$\dot{|p\left(h_{i}=1|h\right)=\sigma \left(c_{i}^{\text{'}}+W_{i}x\right)}\text{and }p\left(x_{j}=1|h\right)=\sigma \left(b_{j}+W_{j}\bar{x}\right)$ are derived for binary units such as *x* ∈ {0,1}^*J*^ and *h* ∈ {0,1}^*T*^. The sigmoid function is indicated as *σ*(). In this way, RBM with binary units is a sigmoid activation function unsupervised neural network. RBM model may be calibrated by reducing negative log-likelihood by gradient descent. RBM takes use of the above conditional probabilities, which makes using a Gibbs sampling approach to generate model samples easier. Gibbs sampling is made much easier by contrastive divergence (CD): (1) begin a Markov chain with training samples and (2) after *k* steps, halt to get samples. It has been demonstrated that a CD with a few steps functions well.

DBN using a training method that trains one layer at a time in a greedy manner. The joint distribution is defined by eq. for visible unit *x* and *l* hidden layers (26)

(26) $$\begin{array}{c} p\left(x,h^{1},\ldots ,h^{\ell }\right)=p\left(h^{\ell -1},h^{\ell }\right)\left(\prod_{k=1}^{l-2}p\left(h^{k}|h^{k+1}\right)\right)p\left(x|h^{1}\right). \end{array}$$

Training at every layer of FDBN is the same as training an RBM since every DBN layer is built as an RBM. Initializing a network using FDBN training is used to undertake classification. An optimization issue is required for each phase. At each layer *k*, the pretraining phase solves optimization issues with the training data set *D*={(*x*^(*l*)^, *y*^(*l*)^,…, *x*^(|*D*|)^, *y*^|(*D*)|^)} having *x* and *y* parameters represented in (27)

(27) $$\begin{array}{c} \underset{\theta_{k}}{\text{min}}\frac{1}{\left|D\right|}\sum_{i=1}^{\left|D\right|}\left[-\text{log }p\left(x_{k}^{\left(i\right)};\theta_{k}\right)\right], \end{array}$$

where *x*_*k*_^(*i*)^ is visible input to layer *k* corresponding to input *x*^(*i*)^, *θ*_*k*_=(*W*_*k*_, *b*_*k*_, *c*_*k*_) is RBM model specification that shows visible bias, weights, and hidden bias in energy function, and *θ*_*k*_=(*W*_*k*_, *b*_*k*_, *c*_*k*_) is RBM model specification that indicates visible bias, weights, and hidden bias in energy. It is worth noting that layerwise updating necessitates resolving ℓ of issues from bottom to top concealed layer is represented in (28)

(28) $$\begin{array}{c} \underset{\phi }{\text{min}}\frac{1}{\left|D\right|}\sum_{i=1}^{\left|D\right|}\left[ℒ\left(\phi ;y^{\left(i\right)},h\left(x^{\left(i\right)}\right)\right)\right], \end{array}$$

where loss function is denoted as *L* (). At layer *l*, final hidden features are denoted as *h* and classifier parameter is *φ*. *h*(*x*^(*i*)^)=*h*(*x*_*l*_^(*i*)^) is used for simplicity. As a result, in this example, we teach FDBN to initialize FFN.

For fine-tuning objectives and summing pretraining, the naïve method is used. This model implements an a2-phase training approach at the same time; however, one extra hyperparameter is required to balance the influence of both objectives. FDBN + loss defined in (29)

(29) $$\begin{array}{c} \underset{\theta_{L},\theta_{DE\;N}}{\text{min}}E_{y,x}\left[ℒ\left(\theta_{L};y,h\left(x\right)\right)\right]+\rho E_{x}\left[-\text{log}p\left(x;\theta_{DB\;N}\right)\right]. \end{array}$$

Empirically based on training samples *D*, as shown in (30),

(30) $$\begin{array}{c} \underset{\theta_{L},\theta_{DB\;N}}{\text{min}}\frac{1}{\left|D\right|}\sum_{i=1}^{\left|D\right|}\left[ℒ\left(\theta_{L};y^{\left(i\right)},h\left(x^{\left(i\right)}\right)\right)-\rho \text{log }p\left(x^{\left(i\right)};\theta_{DB\;N}\right)\right], \end{array}$$

where underlying parameters are *θ*_*L*_, *θ*_*DB*  *N*_, *θ*_*L*_=∅ from (1), and *θ*_*DB*  *N*_=(*θ*_*K*_)_*K*=1_. The hidden space is classified using this approach. It should be more resilient, as it reduces anticipated loss and therefore gives greater accuracy on data not observed. Mathematical designs that reduce anticipated loss function to its smallest value are represented in (31)

(31) $$\begin{array}{c} \underset{\theta_{L},\theta_{DB\;N}}{\text{min}}E_{y,h|x}\left[ℒ\left(\theta_{L};y,h\left(\theta_{DB\;N};x\right)\right)\right]. \end{array}$$

It is empirically based on training samples *D*, as shown in (32),

(32) $$\begin{array}{c} \underset{\theta_{L},\theta_{Da\;N}}{\text{min}}\frac{1}{\left|D\right|}\sum_{i=1}^{\left|D\right|}\left[\sum_{h}p\left(h|x^{\left(i\right)}\right)ℒ\left(\theta_{L};y^{\left(i\right)},h\left(\theta_{DB\;N};x^{\left(i\right)}\right)\right)\right]. \end{array}$$

With notation *h*(*θ*_*DB*  *N*_; *x*^(*i*)^=*h*(*x*^(*i*)^)  based on the dependency of *h* on *θ*_*DB*  *N*_. There are two advantages to this model. First, by limiting parameters fitted in an unsupervised way, the model maintains a decent input. The restriction also helps keep parameters in check by securing them from exploding while being updated.

The mathematical form of model FDBNN classifies given training samples *D* as shown in (33)

(33) $$\begin{array}{c} \underset{\theta_{L,}\theta_{D}\Delta N}{\text{min}}\frac{1}{\left|D\right|}\sum_{i=1}^{\left|D\right|}\left[\underset{h}{\sum }p\left(h|x^{\left(i\right)}\right)ℒ\left(\theta_{L};y^{\left(i\right)},h\left(\theta_{DB\;N};x^{\left(i\right)}\right)\right)\right]\text{s.t.}\left|\theta_{DB\;N}-\theta_{DB\;N}^{*}\right|\leq \delta . \end{array}$$

Optimal DBN parameters are *θ*_*DB*  *N*_^*∗*^ and hyperparameter *δ*. To obtain DBN fitted parameters, this method requires a pretraining phase. It is defined in (34)

(34) $$\begin{array}{c} \underset{\theta_{L},\theta_{DE\;N}}{\text{min}}\frac{1}{\left|D\right|}\sum_{i=1}^{\left|D\right|}\left[\sum_{h}p\left(h|x^{\left(i\right)}\right)ℒ\left(\theta_{L};y^{\left(i\right)},h\left(\theta_{DB\;N};x^{\left(i\right)}\right)\right)\right]\text{s.t.}\left|\theta_{DB\;N}-\theta_{DB\;N-OPT}^{*}\right|\leq \delta , \end{array}$$

where *θ*_*DB*  *N*−*OPT*_^*∗*^ are optimal values of FDBNN specifications after 2 phase training and hyperparameter *δ*. A mathematical model of FDBNN based on *D* training samples is given by (35)

(35) $$\begin{array}{c} \underset{L_{L}\theta_{Dล\;N}}{\text{min}}\frac{1}{\left|D\right|}\sum_{i=1}^{\left|D\right|}\left[ℒ\left(\theta_{L};y^{\left(i\right)},h\left(\theta_{DB\;N};x^{\left(i\right)}\right)\right)\right],\\ \text{s.t.}\left|\theta_{DB\;N}-\theta_{DB\;N}^{*}\right|\leq \delta ,\\ \text{s.t.}\left|\theta_{DB\;N}-\theta_{DB\;N-OPT}^{*}\right|\leq \delta ,\\ \text{s.t.}\theta_{DB\;N}^{*}=\underset{\theta_{DB\;N}}{\arg\text{min}}Z_{x}\left[-\text{log }p\left(x;\theta_{DB\;N}\right)\right],\\ \underset{D_{L},\theta_{Da\;N}^{*}}{\text{min}}E_{y,x}\left[ℒ\left(\theta_{L};y,h\left(\theta_{DB\;N}^{*};x\right)\right)\right]. \end{array}$$

And empirically based on training samples by (36),

(36) $$\begin{array}{c} \underset{\theta_{L},\theta_{Da\;N}^{亡}}{\text{min}}\frac{1}{\left|D\right|}\sum_{i=1}^{\left|D\right|}\left[ℒ\left(\theta_{L};y^{\left(i\right)},h\left(\theta_{DB\;N};x^{\left(i\right)}\right)\right)\right],\\ s.t.\theta_{DB\;N}^{*}=\underset{\theta \text{DaN}}{\arg\text{min}}\frac{1}{\left|D\right|}\sum_{i=1}^{\left|D\right|}\left[-\text{log }p\left(x^{\left(i\right)};\theta_{DB\;N}\right)\right],\\ s.t.\nabla_{\theta_{DB\;N}}E_{x}\left[-{\text{log }p\left(x;\theta_{DB\;N}\right)|}_{\theta_{DB\;N}^{*}}\right]=0. \end{array}$$

With quadratic penalty function, the constrained issue is transferred into unconstrained issue represented in (37)

(37) $$\begin{array}{c} \overset{n}{\underset{\theta_{L},\theta_{Da\;N}}{\text{min}}}E_{y,x}\left[ℒ\left(\theta_{L};y,h\left(\theta_{DB\;N}^{*};x\right)\right)\right]+\frac{\mu }{2}\left\|\nabla_{\theta_{DB\;N}}E_{x}{\left[-\text{log }p\left(x;\theta_{DB\;N}\right)\right]}_{\theta_{Ds\;N}^{*}}\right\|{}^{2}. \end{array}$$

Hyperparameter is denoted as *µ*.

## 4. Performance Analysis

This section discusses the parametric analysis for proposed lung cancer detection by histopathological image analysis. Implementation was done in Python tool, and configurations considered for simulation are PC with Ubuntu, 4 GB RAM, and Intel i3 processor.

Parametric metrics:

Parametric metrics considered for this evaluation consist of accuracy, precision, recall, and F1-Score.

*Accuracy:* It is the ratio of correctly predicted values to the total number of predictions, which is represented in (38)

(38) $$\begin{array}{c} \text{Accuracy}=\frac{\text{TP}+\text{TN}}{\text{TP}+\text{TN}+\text{FP}+\text{FN}}\mathrm{ɛ.} \end{array}$$

*Recall or Sensitivity:* It is ratio of corrected to total predicted value, which is represented in (39)

(39) $$\begin{array}{c} \text{Recall}=\frac{\text{TP}}{\text{TP}+\text{FN}}. \end{array}$$

*Precision*: It is the ratio of TP to total predicted values, which is represented in (40)

(40) $$\begin{array}{c} \text{Precision}=\frac{\text{TP}}{\text{TP}+\text{FP}}. \end{array}$$

*F1-Score:* It is the ratio of the average of precision and recall, which is represented in (41)

(41) $$\begin{array}{c} F1-\text{Score}=2*\frac{\text{Precision}*\text{Recall}}{\text{Precision}+\text{Recall}}. \end{array}$$

Data set description:

LZ2500 (Lung and Colon Cancer Histopathological Image Data set): From the LZ2500 data set, 15000 digital images of histopathological slides are utilized. There are 3 classes of digital pathology pictures from the lungs: class I has 5000 photographs of benign lung tissue, class II contains 5,000 images of lung squamous cell carcinoma, and class III consists of 5,000 images of lung adenocarcinoma.

NLST (National Lung Screening Trial) data set: From original high-resolution histopathological images, 215 tiles of size 512 × 512 are selected. A well-trained pathologist manually weakly annotates the nuclei in these tiles. This data set contains 83245 nuclei objects.

NCI Genomic Data set: From this source, all freely available lung cancer images are uploaded. Using 459 and 1175 eosin-stained histopathology images, automatic classification of the primary tumor and solid tissue normal was studied. Using a set of 567 and 608 whole-slide images, a primary tumor is classified into LUSC and LUAD.

Table 1 shows the histopathological image analysis for the proposed KPCA–CNN_FDBNN and existing techniques compared are 3D CNN [6], and FCN [20].

**Figure 2:**
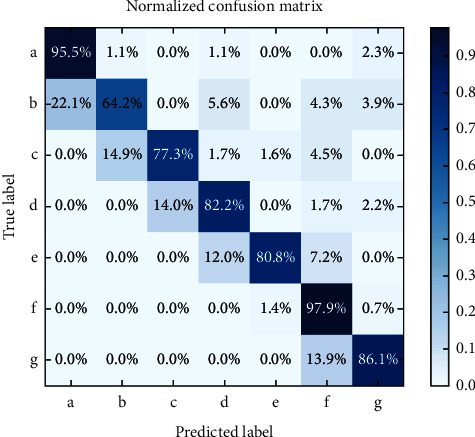
Confusion matrix for cancer detection.

Figure 2 shows the cancer detection confusion matrix. The confusion matrix is evaluated for the actual class as well as the predicted class based on matrix normalization utilizing the proposed KPCA–CNN_FDBNN.

The parametric analysis has been carried out in terms of accuracy, precision, recall, and F-measure. The proposed technique is KPCA–CNN_FDBNN, and the existing techniques compared are 3D CNN [6] and FCN [20].  Table 2 shows a comparative analysis between proposed and extant techniques. A graphical representation for the above table is shown below.

**Figure 3:**
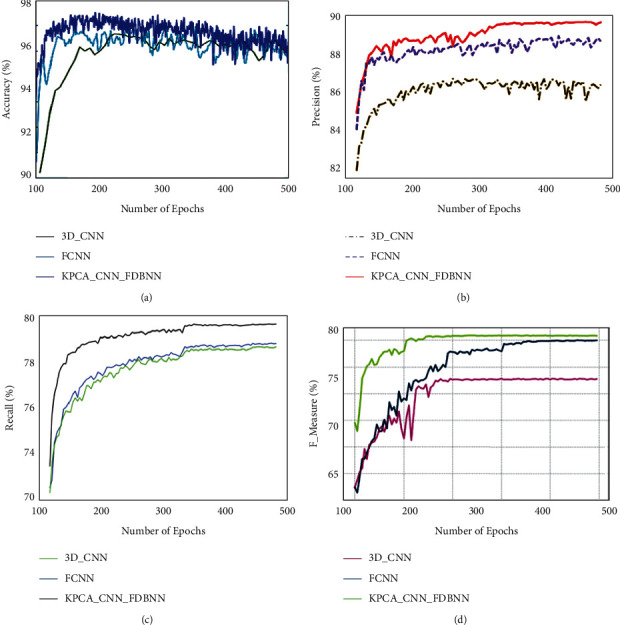
Comparative analysis for LZ2500 data set for (a) accuracy, (b) precision, (c) recall, and (d) F-measure.

Figure 3 shows a comparative analysis of data set LZ2500 in terms of accuracy, precision, recall, and F-measure. A graph has been plotted for a number of epochs with the parameter percentage. Based on this comparison of the LZ2500 data set, the proposed technique obtained an accuracy of 97.1%, 89.9% precision, 79.9% recall, and 79.8% F-measure. These obtained results of the proposed method show optimal results for LZ2500 histopathology image analysis.

**Figure 4:**
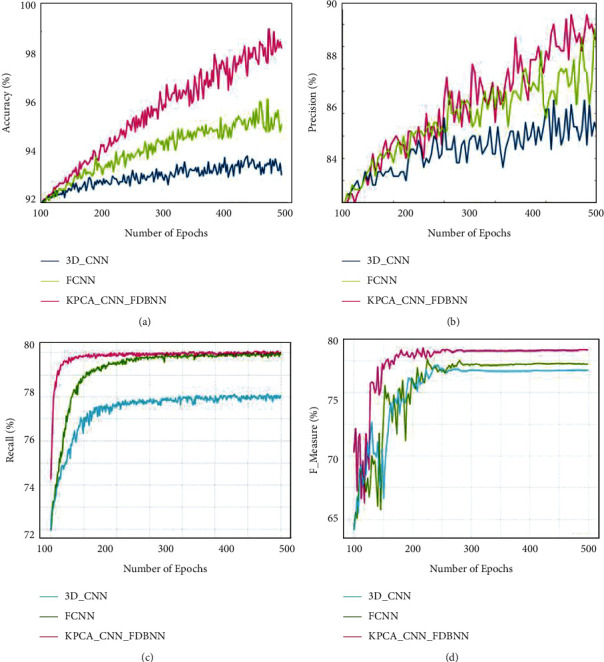
Comparative analysis for NLST data set for (a) accuracy, (b) precision, (c) recall, and (d) F-measure.

Figure 4 shows a comparative analysis of data set NLST in terms of accuracy, precision, recall, and F-measure. From this histopathology image for the proposed technique obtained an accuracy of 98%, 89.1% precision, 79.9% recall, and 79.8% F-measure. The proposed technique obtained enhanced results in the detection of lung cancer from histopathology images from NLST data set.

**Figure 5:**
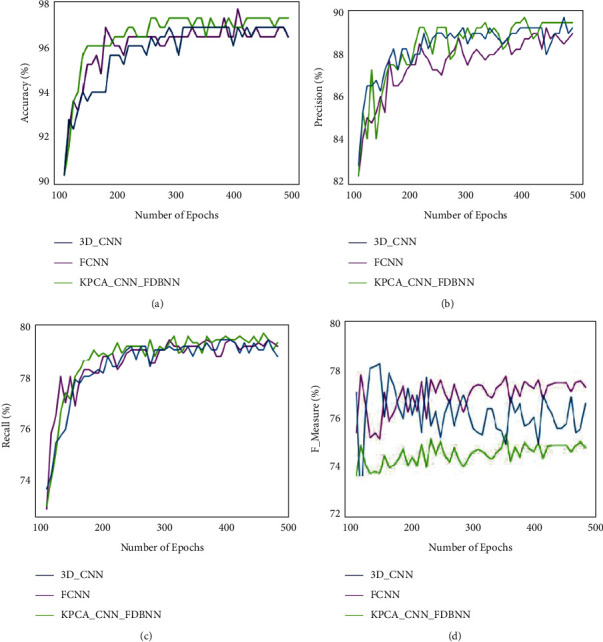
Comparative analysis for NCI Genomic data set for (a) accuracy, (b) precision, (c) recall, and (d) F-measure.

Figure 5 shows a comparative analysis of various parameters for the NCI Genomic data set, which analyses the histopathological image for the detection of lung cancer. The comparative analysis has been carried out in terms of accuracy, precision, recall, and F-measure. From the above analysis, accuracy obtained is 97.5%, the precision obtained is 89.5%, recall of 79.8% has been obtained, and F-measure of 77.9%.

## 5. Conclusion

This paper proposed lung cancer detection based on histopathological image analysis using deep learning architectures. The main contributions carried out in this research are processing the input histopathology image for noise removal, image resizing, and normalizing the image. Then, extracting features for a processed image using KPCA-CNN in which KPCA has been used in the feature extraction layer of CNN and classifying extracted features using FDBNN. The classified output shows the normal cell and cancerous cells of the input histology image. The simulation analysis has been executed for various data sets like LZ2500, NLST, and NCI Genomic data sets for histopathology images for parametric analysis in basic terms and conditions of accuracy, precision, recall, and F-measure. Comparison to the LZ2500 data set, the proposed technique obtained an accuracy of 97.1%, 89.9% precision, 79.9% recall, and 79.8% F-measure. NLST data set obtained an accuracy of 98%, 89.1% precision, 79.9% recall, and 79.8% F-measure, and in the comparative analysis of various parameters for NCI Genomic data set, the accuracy obtained is 97.5%, the precision obtained is 89.5%, recall of 79.8% has obtained, and F-measure is 77.9%.

## Figures and Tables

**Table 1:** Histopathological image analysis.

Input data set	Preprocessed image	3D CNN [6]	FCN [20]	KPCA –CNN_FDBNN
LZ2500	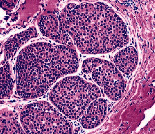	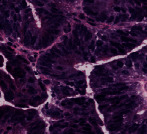	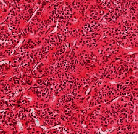	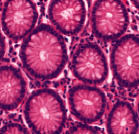
NLST	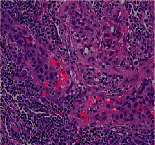	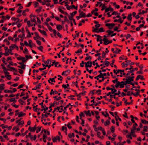	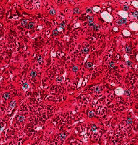	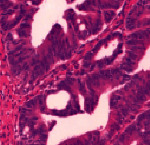
NCI genomic	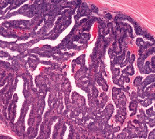	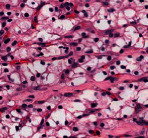	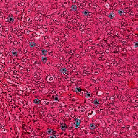	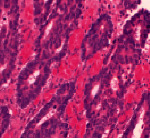

**Table 2:** Comparative analysis of proposed technique with existing methods.

Data sets	Techniques	Accuracy (%)	Precision (%)	Recall (%)	F-measure (%)
LZ2500	3D CNN [6]	95.5	86.2	78.8	74.5
	FCN [20]	96	88.5	79.1	78.9
	KPCA –CNN_FDBNN	97.1	89.9	79.9	79.8
NLST	3D CNN [6]	93.8	86.1	78	77.8
	FCN [20]	96	88.5	79.6	78.1
	KPCA –CNN_FDBNN	98	89.1	79.9	79.8
NCI genomic	3D CNN [6]	96.5	88.6	79	75.1
	FCN [20]	96.8	89.3	79.4	76.5
	KPCA –CNN_FDBNN	97.5	89.5	79.8	77.9

## Data Availability

The data that support the findings of this study are available from the corresponding author upon request.

## References

[1] Hu Z., Tang J., Wang Z., Zhang K., Zhang L., Sun Q. (2018). Deep learning for image-based cancer detection and diagnosis− A survey. *Pattern Recognition*.

[2] Deng S., Zhang X., Yan W. (2020). Deep Learning in Digital Pathology Image Analysis: A Survey. *Frontiers of Medicine*.

[3] AlZubaidi A. K., Sideseq F. B., Faeq A., Basil M. Computer aided diagnosis in digital pathology application: review and perspective approach in lung cancer classification.

[4] Srinidhi C. L., Ciga O., Martel A. L. Deep neural network models for computational histopathology: a survey. *Medical Image Analysis*.

[5] Coudray N., Ocampo P. S., Sakellaropoulos T. (2018). Classification and mutation prediction from non–small cell lung cancer histopathology images using deep learning. *Nature Medicine*.

[6] Li, Zhang J., Zhang T. (2020). Deep learning methods for lung cancer segmentation in whole-slide histopathology images-the acdc@ lunghp challenge 2019. *IEEE Journal of Biomedical and Health Informatics*.

[7] Han Y., Ma Y., Zhang F. (2021). Histologic subtype classification of non-small cell lung cancer using PET/CT images. *European Journal of Nuclear Medicine and Molecular Imaging*.

[8] Wang S., Dong L., Wang X., Wang X. (2020). Classification of pathological types of lung cancer from CT images by deep residual neural networks with transfer learning strategy. *Open Medicine*.

[9] Kather J. N., Heij L. R., Grabsch H. I. (2020). Pan-cancer image-based detection of clinically actionable genetic alterations. *Nature cancer*.

[10] Šarić M., Russo M., Stella M., Sikora M. CNN-based method for lung cancer detection in whole slide histopathology images.

[11] Rahman M. S., Shill P. C., Homayra Z. A new method for lung nodule detection using deep neural networks for CT images.

[12] Shanthi S., Rajkumar N. (2020). Lung cancer prediction using stochastic diffusion search (SDS) based feature selection and machine learning methods. *Neural Processing Letters*.

[13] Huang X., Lei Q., Xie T., Zhang Y., Hu Z., Zhou Q. (2020). Deep Transfer Convolutional Neural Network and Extreme Learning Machine for Lung Nodule Diagnosis on CT Images arXiv Preprint. https://arxiv.org/abs/2001.01279.

[14] Moradi P., Jamzad M. Detecting lung cancer lesions in CT images using 3D convolutional neural networks.

[15] Toğaçar M., Ergen B., Cömert Z. (2020). Detection of lung cancer on chest CT images using minimum redundancy maximum relevance feature selection method with convolutional neural networks. *Biocybernetics and Biomedical Engineering*.

[16] Wei H., Yang F., Liu Z. (2019). Application of computed tomography-based radiomics signature analysis in the prediction of the response of small cell lung cancer patients to first-line chemotherapy. *Experimental and Therapeutic Medicine*.

[17] Shin H., Oh S., Hong S. (2020). Early-stage lung cancer diagnosis by deep learning-based spectroscopic analysis of circulating exosomes. *ACS Nano*.

[18] Shanthi S., Rajkumar N. (2021). Lung cancer prediction using stochastic diffusion search (SDS) based feature selection and machine learning methods. *Neural Processing Letters*.

[19] Song Z., Zou S., Zhou W. (2020). Clinically applicable histopathological diagnosis system for gastric cancer detection using deep learning. *Nature Communications*.

[20] Guo Y., Song Q., Jiang M. (2021). Histological subtypes classification of lung cancers on CT images using 3D deep learning and radiomics. *Academic Radiology*.

[21] Yao J., Zhu X., Jonnagaddala J., Hawkins N., Huang J. Whole slide images based cancer survival prediction using attention guided deep multiple instance learning networks. *Medical Image Analysis*.

[22] Kanavati F., Toyokawa G., Momosaki S. (2020). Weakly-supervised learning for lung carcinoma classification using deep learning. *Scientific Reports*.

